# “*Inclusivity requires an active effort*”: building an inclusive and diverse space when engaging people with lived and living experience and caregivers in mental health and substance use health research

**DOI:** 10.1186/s40900-025-00798-w

**Published:** 2025-10-29

**Authors:** Abigail Amartey, Shoshana Hauer, Charlotte Munro, Claudia Sendanyoye, Katie Upham, Mary Rose van Kesteren, Tanya Halsall, Yona Lunsky, Lisa D. Hawke

**Affiliations:** 1 Centre for Addiction and Mental Health, Toronto, Canada; 2 Department of Psychiatry, University of Toronto, Toronto, Canada; 3 University of Ottawa Institute of Mental Health Research, Ottawa, Canada

**Keywords:** Patient-oriented research, Patient engagement, Lived experience, Diversity, Inclusivity

## Abstract

**Background:**

Engaging people with lived/living experience and caregivers (PLLEX-C) in mental health and substance use health research has many advantages for scientists, research staff, and PLLEX-C. However, research teams must be cognizant of the wide spectrum of human diversity. As such, engagement settings that are inclusive and reflect the diversity of the populations being served are essential to meaningful and impactful research.

**Objective:**

The aim of this qualitative descriptive study was to understand the perspectives of PLLEX-C on how to build inclusive and diverse research spaces when engaging PLLEX-C in mental health and substance use health research.

**Methods:**

We recruited 20 PLLEX-C with experience engaging in mental health and substance use health research to participate in one of five focus group discussions. The focus groups were audio recorded and transcribed, with codebook thematic analysis conducted using a deductive and inductive approach. This study team included a Lived and Living Experience and Caregiver Working Group throughout all phases of the research project.

**Results:**

Four themes were identified across the five focus group discussions: 1) Acknowledge that diversity is inclusive of different factors and this needs to be reflected in the recruitment process to improve the research. 2) Remove barriers of entry into the research space. 3) Ensure that the scientists and staff are trained and skilled in inclusive and diverse engagement. 4) Build a safe and equitable engagement space. Within these themes, subthemes were also identified and described with illustrative quotes.

**Conclusions:**

Identifying ways to ensure research engagement settings are inclusive and diverse for all those involved requires an intentional and active effort at all stages of the research process. This includes not only employing various recruitment strategies to identify more diverse PLLEX-C, but also continuous, ongoing training for researchers to ensure engagement is culturally sensitive, anti-discriminatory, and bias-free. Prioritizing research teams that are inclusive and diverse can foster an engagement experience that is more meaningful, authentic, and empowering.

**Supplementary Information:**

The online version contains supplementary material available at 10.1186/s40900-025-00798-w.

## Abstract (plain language summary)

In mental health and substance use health research, we see many benefits when people with mental health and/or substance use health experiences and challenges are included on research teams. They are referred to as ‘people with lived/living experience and caregivers’ (PLLEX-C). We wanted to understand how to create a more inclusive, diverse space for the people engaged in these roles. We held five group discussions with PLLEX-C who have previously been members of mental health and substance use health research teams. They were asked to share their thoughts on how research teams can include PLLEX-C from more diverse backgrounds. Participants said that diversity is made up of different characteristics that should be included on teams; that research spaces need to be free of barriers that prevent individuals from taking part in the research; that scientists and research staff must receive diversity and inclusion training; and that the research space must make everyone feel safe and equal to others. Mental health and substance use health research teams need to make an active effort to include PLLEX-C from more diverse backgrounds as people’s health and their care experiences will vary across socioeconomic factors, ethnicity, gender, age, and other identity factors. This will lead to more people being included in the research process, and will lead to higher quality research.

**Supplementary Information:**

The online version contains supplementary material available at 10.1186/s40900-025-00798-w.

## Introduction

In recent years, it has become increasingly common to engage people with lived and living experience and caregivers (PLLEX-C) in mental health and substance use health research, also known as patient-oriented research or lived experience engagement in research [1]. PLLEX-C function in a variety of roles such as *advisors* (providing advice or guidance while often sitting on an advisory group), *co-researchers* (equal partnership with academic researchers to engage in some or all research activities), *collaborators* (ongoing partnership providing help on different project activities), and *co-producers* (sharing power and responsibility with academic researchers from the start to end of a project) bringing with them a wealth of knowledge, skills, and experiences that make them valuable members of the research team [2–7]. This leads to significant contributions from PLLEX-C, such as guiding research questions, co-developing research design and methodology, analyzing data and interpreting results, and co-authoring research papers and other knowledge translation products [4, 8]. Meaningful engagement where PLLEX-C perspectives are sought after and included in every step of the research process leads to data that is purposeful, relevant, and more reflective of their unique lived/living experiences [7, 9].

An important aspect of engagement in mental health and substance use health research is the effective recruitment and retention of an inclusive, diverse set of PLLEX-C team members. However, common barriers and facilitators emerge in relation to logistics of recruitment and retention, such as technological barriers [10], intrapersonal factors [11–13], team dynamics [12–14], communication [13–16], knowledge and attitudes [17], planning and implementation [13, 15], and institutional factors [12, 14, 15, 18, 19]. A lack of diversity among PLLEX-C team members has also been highlighted as a barrier to successful engagement at the planning and implementation phase [7, 13, 14, 20]. Moreover, a recent scoping review found diversity and inclusivity to be a major research and implementation gap in engaging PLLEX-C in mental health and substance use health research [21]. This has led some researchers to express concern that PLLEX-C engagement does not reflect the diversity of the populations their research aims to serve, and instead represents members of the population that are easier to engage with—thus leading to further marginalization [22–25]. To advance the understanding of successful and productive engagement, it is essential to address barriers and gaps in research evidence and implementation related to diversity and inclusivity at all levels of research.

Researchers and PLLEX-C involved in research emphasize the need to increase the diversity of perspectives among PLLEX-C team members by engaging individuals with a broader range of lived experiences. While different descriptions for diversity exist, the current article focuses on “variety diversity”, which considers differences in group population based on specific categorical variables [26]. This includes age, race and ethnicity, disability, Indigeneity, gender identity, and sexual orientation, among other factors that shape health equity [15, 27–30]. Engaging these communities will foster a greater understanding of the perspectives of equity-owed populations by reconceptualizing the approach of the research being conducted [31, 32]. Furthermore, it helps support equitable opportunities and centres the voices of the individuals who are targeted by the research and are therefore most affected by the research being conducted [33]. While some research suggests actively working to minimize power imbalances [15] and allowing flexible ways to engage [23, 24] to achieve more diversity, little is known about how PLLEX-C view efforts to increase diversity and inclusivity, particularly in mental health and substance use health research spaces, where PLLEX-C voices have historically, persistently, and systemically been excluded [34]. Rich descriptions of how PLLEX-C experience diversity and inclusivity within the research engagement space, including barriers and facilitators, is an area that requires further exploration.

### Objective

To understand the perspectives of PLLEX-C regarding how to build an inclusive and diverse space when engaging people with lived/living experience and caregivers in mental health and substance use health research.

## Method

We engaged PLLEX-C throughout all phases of this study utilizing the Patient Engagement in Research framework [35]. The overall study is described using the Standards for Reporting Qualitative Research (SRQR) reporting guidelines found in Appendix A [36], while the engagement process is described in Appendix C using the Guidance for Reporting Involvement of Patients and the Public-2 (GRIPP-2) Checklist [37]. Finally, a pragmatist paradigm was employed, which has previously been identified as an optimal guiding approach to use in patient-oriented research [33].

### Participants & recruitment

PLLEX-C from across Canada were recruited using purposive sampling [38] to participate in one of five focus groups. Eligibility criteria included individuals aged 16 years of age and older, who were residents of Canada, and who had been previously engaged in mental health and/or substance use health research as a person with lived or living experience or family/caregiver as advisors, collaborators, co-researchers or in another similar role that did not involve only being a past research participant.

All study procedures were conducted at the Centre for Addiction and Mental Health, a mental health teaching hospital and research centre with an organizational commitment to embracing PLLEX-C engagement. We recruited participants by inviting individuals from across Canada who previously participated in any of four engagement-related online research projects led out of the same institution. We also posted our study flyer nationally on the institutional social media (X) account, which was shared by engagement-related organizations. By using the database of previous participants from across Canada, we were able to target potential participants representing diverse populations.

### Procedure

Research staff screened and obtained informed consent from interested prospective participants. Upon providing signed consent, participants completed a demographic form on REDCap [39], a web application hosted on a secure server. Participants were then invited to the next scheduled focus group. The focus group discussions were held from January 23rd to February 19th, 2025, virtually on the WebEx video conferencing platform, hosted on our secure institutional server. The focus group meetings were audio recorded using WebEx and a handheld recorder; discussions were transcribed by the WebEx transcription system, and subsequently cleaned by research staff members. The five focus groups were co-facilitated by one research staff member and one research placement student, and lasted between 71 and 96 min. In total, 20 people participated in the focus group discussions, ranging from 2 to 6 participants in each of the five groups. To facilitate use of the WebEx platform and address accessibility needs, research staff helped troubleshoot any issues related to connectivity and oriented participants to WebEx features (e.g., mute/unmute, hand raise function, etc.). They also enabled the chat function to provide participants with alternative ways to contribute to the discussions based on their preference and comfort level. We provided a $50 gift card to participants. This study received institutional Research Ethics Board approval from the Centre for Addiction and Mental Health.

### Data collection tools

The demographic form contained personal information related to identity (i.e., age, gender, ethnicity, language, country of birth, and education), as well as items related to level of experience engaging in research. A semi-structured interview guide was developed for the focus groups and included a range of discussion points on diversity and inclusivity in spaces engaging PLLEX-C in mental health and substance use health research. A sample question included in the interview guide was, “*People with lived experience and family members tell us that it’s important for research teams to be inclusive. Based on your lived experience, what does inclusivity mean to you?*” A copy of this guide is included in Appendix B. The demographic form and interview guide were drafted by the scientists and staff and refined by a Lived/Living Experience and Caregiver Working Group (PLLEX-C Working Group) consisting of five PLLEX-C team members on the study.

### Data analysis

Codebook thematic analysis was conducted, using a deductive and inductive approach involving preliminary code development [40]. A meeting was held with one co-facilitator, a research staff member who would be doing the data analysis, one PLLEX-C Working Group member, and the lead researcher to guide the initial creation of a codebook through an in-depth discussion of the focus group findings. The data analyst then entered the codes into NVivo12 [41], and coded the focus group transcripts. Codes were iteratively discussed and refined through bi-weekly meetings with the scientist, research staff, and PLLEX-C Working Group as coding progressed. To enhance accuracy and fidelity, two subsequent meetings were held with the PLLEX-C Working Group to review focus group quotes, and continue to iteratively refine the codebook through open discussion. The group collectively identified specific quotes that are used below to illustrate themes and sub-themes.

### Lived/living experience and caregiver engagement

This project was supported by a PLLEX-C Working Group. A description of the engagement process using the Guidance for Reporting Involvement of Patients and the Public-2 (GRIPP-2) Checklist [37] was completed collaboratively with the Working Group, and is described below and in Appendix C.

#### Engagement aim

The PLLEX-C team members were engaged in this study to draw on their lived/living expertise, learned expertise, and knowledge base. Their perspectives helped to close the gap between the research and their experiences of mental health and substance use health, which helped inform our study on how to make research engagement spaces more inclusive and diverse.

#### Engagement methods

A PLLEX-C Working Group was recruited from the institutional network of PLLEX-C interested in research engagement. The Working Group consisted of three to six members, depending on the project stage. At study initiation, the research team developed a terms of reference agreement, and a consensus meeting time was decided by poll. We launched the virtual Working Group meetings in April 2024 and concluded them in July 2025. There were 20 meetings held from the design of the overarching study to the finalization of the current manuscript. Meetings were initially 1 h in duration; however, they were extended to 2 h based on need. They were held either monthly or semi-monthly, with the team working on this project and other related sub-projects. Compensation was provided at an hourly rate for meeting attendance and study-related tasks completed outside of meeting times based on a flexible pre-specified allotment of time for activities. PLLEX-C also indicated their method to receive honoraria. Several accessibility supports were provided, including alternative software options, basic training of the WebEx system, troubleshooting technical difficulties, allowing multiple ways to contribute (e.g., chat, email, or verbal communication/feedback), and allowing Working Group members to participate with their camera off during team meetings.

#### Engagement results

PLLEX-C were engaged at all levels of the research process, from design to knowledge translation. PLLEX-C reviewed research ethics materials (e.g., the interview guide and study flyer). One member helped develop the codebook. All members analyzed initial codes and themes, chose quotes for inclusion, co-authored the manuscript, helped with grant and conference applications/submissions, and suggested relevant research questions for future exploration.

#### Discussion and conclusions of the engagement process

The PLLEX-C engagement approach promoted trust and collaboration. The research was rooted in real-world experience, equity, diversity, and inclusivity. Strategies for recruitment and study tools had greater relevance to the participants. Meetings were initially held on the WebEx system, but were moved to Zoom to accommodate Working Group familiarity and preference. We also held ongoing informal conversations about how engagement was going, which supported iterative improvements to our engagement approach. Engagement was formally evaluated using the Patient Engagement in Research Scale near the end of the project period [42].

#### Reflections and critical perspective on engagement

PLLEX-C were able to build personal and professional skills through their involvement in the Working Group. This included a better understanding of qualitative research methods, as well as learning how to address the needs of the project and interpersonal interactions in a group environment. Their lived and living experiences were respected and valued and their experiences and perspectives were affirmed. Scientists and research staff on the team grew their understanding of the structural components of the work, which facilitated reflection and co-learning.

#### Research team reflexivity

The data analyst (AA) is a black woman with master’s degrees in public health and social work, and is also a Registered Social Worker. This interdisciplinary lens guided her approach to the research process with an emphasis on understanding the importance of cultural context, power dynamics, and the meaning of safe and equitable research spaces. The scientific lead (LDH) is a white woman with a Ph.D. in psychology and a research focus on lived/living experience and caregiver engagement in research. The current study supported a pragmatic approach to informing her commitment to improve diversity and inclusivity in all research spaces engaging PLLEX-C in mental health/substance use health research. The work in this study was also strengthened by the active involvement of co-authors with lived experience, whose perspectives shaped the study design, interpretation, and dissemination. Specifically, the PLLEX-C Working Group consists of experts with a diversity of education, professional experiences, life stories, lived/living and caregiver expertise in mental health and/or substance use, as well as diversity across age, racial/ethnic background, and geographical location. The Working Group members all had experience engaging in research and together with the research staff and scientific lead are dedicated to enhancing the theory and application of engagement research.

## Results

Table 1 presents the characteristics of the focus group participants. Most of the sample were women (65%); however, a diverse mix of participants were seen across age, country of birth, racial/ethnic background, and type of research engagement experience. There were four themes and 14 sub-themes identified from the focus group discussions that are presented in Table 2.

**Table 1:** Sociodemographic and engagement experience of participants

Characteristic		*N* (%)	
Age	16–29	7 (35.0)	
	30–59	6 (30.0)	
	60+	1 (5.0)	
	Missing	6 (30.0)	
Gender	Woman	13 (65.0)	
	Man	5 (25.0)	
	Transgender/gender diverse	2 (10.0)	
Born in Canada	Yes	8 (40.0)	
	No	12 (60.0)	
Racial /ethnic background	South Asian	7 (35.0)	
	White	6 (30.0)	
	Black	4 (20.0)	
	East/Southeast Asian	2 (10.0)	
	Multiple ethnicities	1 (5.0)	
Population of focus of the research engaged on	Child-focused research	5 (25.0)	
	Youth-focused research	16 (80.0)	
	Adult-focused research	12 (60.0)	
	Geriatrics-focused research	5 (25.0)	
Engagement role	PLLEX	11 (55.0)	
	Family/Caregiver	4 (20.0)	
	Both	5 (25.0)	

Note: PLLEX = people with lived or living experiences and challenges of mental health and/or substance use health

**Table 2:** Themes and sub-themes generated from data collected

Theme	Subtheme
1. Acknowledge that diversity is inclusive of different factors and this needs to be reflected in the recruitment process to improve the research.	1a) Different disciplines, job titles, and skill sets
	1b) Race, ethnicity, age, and other identity factors
	1c) Lived/living experiences
	1d) Intentionally recruit diversity to improve research
2. Remove barriers of entry into the research engagement space.	2a) Technology barriers
	2b) Flexible methods to contribute to the research
	2c) Fair and flexible compensation
	2d) Transparency
3. Ensure that the scientists and staff are trained and skilled in inclusive and diverse engagement.	3a) Apply best practices in engagement
	3b) Avoid tokenistic engagement
4. Build a safe and equitable engagement space.	4a) Avoid stigmatizing language, labels, and behaviour
	4b) Use culturally sensitive engagement approaches
	4c) Create an equitable space where everyone is comfortable speaking up
	4d) Communicate clearly

### Acknowledge that diversity is inclusive of different factors and this should be reflected in the recruitment process to improve the research

Participant discussions highlighted that there is no one definition of diversity, and that it holds different meanings for different people (subtheme 1a and 1b). This includes participants describing the diversity of their past research teams as being a mix of different disciplines, professional roles, and educational backgrounds, as well as race, ethnicity, age, and other social identity factors, and lived/living experiences.

One participant raised this point in the context of feeling like a comfortable member of the team within a diverse disciplinary space, stating:

*I find there’s a diverse group of people on those teams. Some of them are medical professions, some you know have other backgrounds, master’s [degrees], and whatever. And they always make you feel like you are part of the group. (Participant 01)*

Although some highlighted this diversity specifically while describing scientists and staff, one participant provided an important reminder that this diversity of disciplines and educational backgrounds also exists amongst PLLEX-C, who can contribute to the research in multiple ways, not just in terms of their lived/living experience: “*There’s a certain reductive attitude of you’re here as a person with [lived/living] experience, and hence that is all that you can contribute” (Participant 02).*

For many participants, diversity in the research space meant ensuring that there is representation from different social identities (subtheme 1b). One participant reflected on how diverse identities can positively impact the research:

*Whenever I think about diversity in the research space, I tend to see the different identities such as age, gender, race, and (…) how it influences the research. And I think that if everyone looked the same or acted the same or had the same beliefs, the research wouldn’t be as fruitful. (Participant 03)*

With that said, some participants highlighted that, in the context of virtual meetings, it was not always possible to see how diverse a team was with respect to more visible identities (e.g., race, age, gender) if cameras were off. However, a variety of lived/living experiences around the research table still helped contribute to a feeling of diversity and richness of perspectives (subtheme 1c), with one participant stating:

*…humans are wired for storytelling, right? And when you include a diverse group of people, you can get a range of stories to whatever concern you’re talking about. And I feel like when you don’t have those different perspectives, you may have the data, but (…) it’s hard for you to know the reality of that condition and the impact it has on people. (Participant 04)*

Finally, there was a general acknowledgement of the complexities of creating diverse research spaces due to a variety of barriers. Participants stressed the importance of needing to be intentional when recruiting for diversity (subtheme 1d). One participant highlighted this intentionality when discussing their own experience:

*I got a chance to basically learn about how the data was going to be analyzed and whether we were over-represented compared to the general population (…) we discussed plans to approach organizations that could help us target some of the populations that we were lacking in our initial recruitment effort. (Participant 04)*

### Remove barriers of entry into the research engagement space

A common theme raised by participants was the existence of barriers that could differentially impact certain groups and make it harder for them to engage in research. Participants discussed removing technology barriers, providing flexible methods to contribute to the research, ensuring transparency, and providing fair and flexible compensation for all to ease entry into the research engagement space.

One barrier in particular—technology—was frequently raised (subtheme 2a). While virtual meetings can remove barriers for those with difficulties travelling to a meeting, for some individuals this can be an added barrier to participation. One participant highlighted the importance of being adaptable when trying to meet with PLLEX-C, stating:

*Are you (…) going to where they are at to get the authentic answers? And that can be applied to so many different communities that don’t have accessible technology. (Participant 05)*

This need for flexibility also extended to the ways in which PLLEX-C can contribute to the research process (subtheme 2b). Participants appreciated having different methods to provide input. One person noted the inclusive nature of this approach when participating in online meetings:

*In some of my studies that I’ve participated in, we’ve had different ways to participate. For example, just even being able to use the chat (…) I think just being able to have different opportunities to be able to share our thinking and ideas for me (…) that is also part of inclusion. (Participant 06)*

Fair and flexible compensation was also raised as an important way to remove barriers of entry to the research engagement space (subtheme 2c). Regarding compensation, participants emphasized the need for teams to be flexible with compensation methods, as highlighted by this participant:

*…you know sometimes engagement also has to do with how do you pay the participants? Like some participants need to be compensated by cash and that’s ok, some people prefer gift cards (…) and some people it’s [gift cards] just not an option. (Participant 09)*

In addition to flexible compensation, fair compensation was also stressed. One participant expressed the lack of inclusivity: “*I really feel not included when I’m in the room with university staff, professors, doctors … but [the] cleaning lady makes much more than I do in that hour”. (Participant 08)*.

Lastly, a lack of transparency was also raised as a barrier that was preventing some individuals from wanting to initiate or continue engaging in research (subtheme 2d). This was particularly seen as an issue in the context of addressing accessibility concerns. One participant spoke of an experience where a visually impaired lived experience partner left their advisory group due to a lack of inclusive engagement by the scientists and staff. Another participant highlighted the need for transparent documentation of this issue:

*…ideally there should be a way to document this, as in people should know that this happened at least, so then there’s ways to improve. Because otherwise the whole thing is just kind of removed from record. It’s almost as if this whole challenge of not being accessible or inclusive never existed. (Participant 07)*

Further strengthening transparency, one participant expressed the value of receiving meeting transcripts to ensure important discussion points are always captured on record. They stated *“It’s verifying to me. It’s showing transparency and respect” (Participant 05)*, indicating that this created a more inclusive environment.

### Ensure that scientists and staff are trained and skilled in inclusive and diverse engagement

Participants shared that to best foster inclusive and diverse research spaces, scientists and staff need to be knowledgeable and well-versed in engagement practices. They must also be skilled at applying best practices in engagement, while avoiding tokenistic engagement of PLLEX-C team members.

Among the best practices in inclusive and diverse engagement (subtheme 3a), participants highlighted the importance of training in effective communication, particularly as it relates to how best to effectively communicate with a diverse range of people.

One participant expressed this as follows:

*How do you effectively talk to a caregiver? What is trauma informed language? What kind of perspective do you need to come from — with great respect to these people that have lived that [experience]. [A]nd what’s an appropriate way to be asking the questions? (Participant 05)*

Tokenistic engagement was also a frequent discussion point (subtheme 3b). Participants underscored the need for PLLEX-C to be meaningfully engaged in the research process, and that scientists and staff must go beyond a superficial level of engagement. A common sentiment was the notion that it may be better to not do engagement than to do poor engagement, with one participant stating:

*[Y]ou do want to avoid (…) tokenism and not including people just for the sake of including people, because what makes me feel worse — or maybe just as bad as not being included is being put on something and you’re just there to check a box. (Participant 11)*

This shows that researcher training and readiness to engage diverse people authentically is important, but equally important is an explicit effort to avoid tokenism to build inclusive and diverse engagement spaces.

### Build a safe and equitable engagement space

Participants described a key belief that if the research engagement of PLLEX-C is to be inclusive, then scientists and staff must cultivate a safe and equitable space where people are comfortable being themselves and speaking freely. This involved a variety of factors, such as avoiding stigmatizing language, labels and behavior, using culturally sensitive engagement approaches, creating an equitable space where everyone is comfortable speaking up, and communicating clearly.

One way to cultivate a safe and equitable space is to create a space free of stigmatizing language, labels, and behaviours—a space where PLLEX-C members do not feel othered (subtheme 4a). One participant stated this desire to “*break through those walls of stigma” (Participant 10)* in the research space. Another participant described the feeling as:

*…people with lived experience as an outgroup to the research team while being within the research team (…) As in there is a very ‘us and them’ narrative (…) you have to prove yourself to be taken seriously. (Participant 02)*

Participants also emphasized the need to use culturally sensitive engagement approaches (subtheme 4b). This takes into consideration how intersecting social identities such as race, gender, and disability shape PLLEX-C experiences of marginalization and their engagement with research. These identities do not exist in isolation; rather, they interact with broader structures of power, privilege, and oppression, influencing how persons are included, excluded, or positioned in research processes. One participant underscored the importance of applying this lens to inclusive engagement practices, saying:

*I feel that inclusivity requires an active effort to ensure that perspectives are viewed from an intersectional lens. (Participant 12)*

Furthermore, this view and understanding of culturally sensitive engagement approaches was seen as crucial even before engaging with particular populations to avoid potential conflict. Using alcohol research as an example, one participant stressed being mindful when reaching out to certain community groups to increase your diversity, as many people may not consume alcohol for cultural reasons: *“[I]f you’re not aware of the populations that you’re reaching out to in terms of the research, it can (…) cause conflict or tension.” (Participant 13).*

In addition, participants shared that it was not enough to just bring PLLEX-C to the table, but that they must be given the “*tools that they need in order to be on the same playing field as everyone else” (Participant 14)* in order to feel comfortable sharing their opinions in the group in an inclusive manner (subtheme 4c).

It was also important that this feeling be created early in the engagement process:

*You are not the accessory or the add-on as someone with lived experience. You’re a contributor from the get go and you’re treated equal to everyone else around the table. (Participant 01)*

Lastly, clear communication was highly valued as a means of fostering a safe and equitable engagement space that fosters inclusivity (subtheme 4d). Participants encouraged scientists and staff to avoid using jargon and acronyms, and to also include and invite PLLEX-C into the conversation as stated by one participant:

*[Y]ou have different providers or researchers or people that are there and they’re having all these conversations at a higher level and totally ignoring you (…) They’re not talking to you, they’re talking at you and around you and over you. (Participant 05)*

This shows that building a safe and equitable engagement space can help foster inclusivity and diversity in a number of ways, helping to make sure that the PLLEX-C at the table can contribute authentically from their unique perspective.

## Discussion

This qualitative descriptive study examined PLLEX-C perspectives on how to build an inclusive and diverse space in the context of lived/living experience and caregiver engagement in mental health and substance use health research. Participants emphasized the importance of recognizing that diversity encompasses a range of factors, and that this should be intentionally reflected in the recruitment process to enhance the quality and relevance of the research. They also underscored the importance of removing barriers of entry into the research engagement space, and ensuring that research teams receive appropriate training in inclusive, equity-oriented, and culturally sensitive engagement practices. In addition, participants highlighted the need to cultivate safe and equitable environments that support inclusive and diverse engagement. Collectively, these recommendations are essential to advancing more inclusive and diverse research engagement.

When thinking about inclusion and diversity, researchers should think broadly about the wide range of factors that make people unique and the range of voices they want at the table, then intentionally work to recruit PLLEX-C representing those voices to the table. One way to do this is for researchers to consider asking PLLEX-C of diverse backgrounds to help identify other diverse PLLEX-C for their research teams. PLLEX-C might be connected to other people in their networks and can help identify those with different characteristics than those already at the table. Research has shown that this form of participant recruitment, known as snowball sampling, has been successful in recruiting harder to reach populations to research [43, 44], and might also be extended to the recruitment of PLLEX-C team members. Another important strategy is outreach to organizations and other community stakeholders that represent specific equity-owed populations [25, 45].

In doing so, it is important for researchers to discuss what PLLEX-C need to be meaningfully engaged in a collaborative way, as this might help them identify barriers that may hinder full inclusivity. By addressing a range of barriers to entry into the engagement space, it is possible to create more inclusive and diverse engagement settings, which stands to improve the research process and outputs. For example, technology-related challenges can be addressed proactively through early identification of access needs, accessibility supports and thoughtful budgeting [46]. This includes being prepared to adapt to technological challenges, or offering alternative options like in-person or telephone call if videoconferencing is not possible for the participant [46]. This approach helps ensure that engagement is not only inclusive, but also equitable and accessible.

Moreover, understanding the structural barriers that exist in engaging with mental health and substance use health services more broadly is also critical. Certain equity-owed groups experience intersecting forms of oppression, including racism, colonialism, sanism, ableism, and economic marginalization, that shape how they access and are treated within mental health and substance use health systems [47–49]. Among those who have engaged in research, suspicion and distrust of researchers due to unethical practices, lack of transparency, or other negative experiences have historically been reported amongst people with mental health and substance use health experiences and challenges, racial/ethnic minority communities, and Indigenous populations [50–55]. As a result, these experiences can influence PLLEX-C’s willingness to engage in research. 

It is important to consider these barriers before recruiting new PLLEX-C, including prior to reaching out to community groups and organizations. Some mitigating strategies that have been suggested include establishing trust at the start of the research process by holding in-person meetings for those who find it supportive [24, 56, 57], allowing flexible ways to engage [8, 23, 24], and providing clear role definition for PLLEX-C [57, 58]. Additional strategies derived from literature focused on recruiting diverse research participants, and may be applicable to identifying more diverse PLLEX-C, include research staff exercising cultural humility and safety, staff who are bi- or multilingual, offering financial support and honoraria, employing targeted recruitment strategies, and providing clear and thorough explanations of research procedures [53]. Research teams must also be disability informed and gender competent. By addressing these barriers internally before doing active outreach, it might be possible to make the space more inclusive and welcoming to diverse PLLEX-C.

In this study, participants identified researcher training as an important factor to ensure diversity and inclusivity. Institutions might consider requiring that researchers obtain training around engagement, including guiding principles and benefits, skills and how to apply best practices, as well as diversity before beginning the engagement process [59–61]. Co-developed training modules that reflect the expertise and needs of PLLEX-C should also be made available to researchers and research staff. A scoping review on patient-oriented research competencies identified the need for researchers to be knowledgeable of the communities they engage with, and to understand how to conduct research within the cultural perspectives of these groups [61]. Therefore, training modules should go beyond basic introductions to research engagement, and include modules on trauma-informed engagement, using non-stigmatizing language, cultural sensitivity, anti-oppression, anti-stigma, diversity, and inclusion. Furthermore, researcher training that identifies and addresses unconscious biases is extremely important, as researchers might be unaware of the biases they hold towards issues related to PLLEX-C, mental illness, substance use health, social and structural determinants of health, and specific cultural and ethnic backgrounds. Importantly, the training should be a continuous, ongoing process, rather than a one-time or ad hoc training.

Lastly, focus group participants emphasized the need to build a safe and equitable space for inclusive and diverse engagement. As teams diversify, an increase in various perspectives might lead to more challenging interactions. It is important to note that conflict or misunderstandings do occasionally occur in interpersonal spaces and this will inevitably occur in engagement processes periodically [12–14, 30]. Building a safe and equitable space for diverse and inclusive engagement therefore requires providing a space where PLLEX-C can raise concerns and mitigate challenges in a constructive and transparent way. Establishing a process to address challenges may include having a neutral third party available to provide support and troubleshooting. Other co-produced mitigation strategies might be considered. In addition, tokenism, in which PLLEX-C are superficially engaged by researchers for optics (i.e., “ticking a box”) should be avoided to not only foster a safe and equitable space, but to maximize meaningful contributions [12, 15, 19]. This is particularly important when engaging individuals facing systemic forms of oppression that already function as a barrier to engaging in research [62]. Power differences can also exist amongst PLLEX-C who bring a wealth of experience, knowledge, skills, and perspectives to research teams [63]. Researchers should be mindful of any implicit biases they may hold towards certain viewpoints, and maintain a research space that values all PLLEX-C contributions.

This study has strengths and limitations to keep in mind.

### Strengths

Strengths of this study include co-production with a PLLEX-C working group as part of the research team. The regular frequency of meetings and in-depth engagement in the study was a strength. The research team was diverse in terms of age, lived experience, and ethnic/cultural diversity, dis/ability, although all were women. The study sample contained substantial ethnic and cultural diversity. However, certain subgroups of the population were either unrepresented, under-represented, or unknown as the identity factor was not asked about, such as Indigenous Peoples, homeless or under-housed people, transgender individuals, and substance use health specific populations.

### Limitations

Limitations include a majority of women participants, reflecting a diversity gap. We did not collect data on whether participants were engaged in mental health versus substance use health research, which might have left some unanswered questions. Future research should examine the needs of specific groups, or engage in larger data collection with case analysis across different diversity groups. Limited study funding prevented us from pursuing more of this work. Due to challenges with recruitment, we gathered a smaller number of participants for each of our five focus groups (i.e., median: 5 participants per group). This is described in the literature as “mini focus groups” consisting of two to five participants [64]. While small focus group sizes risks limiting the depth of qualitative data collected [65], other researchers believe smaller groups are easier to manage and can lead to greater interaction and discussion [66, 67].

## Conclusions

This study identified PLLEX-C perspectives on ways to ensure that PLLEX-C research engagement spaces are inclusive for all those involved, across a wide range of diversity factors, by breaking down the barriers of entry into engagement, training the researchers and research staff on authentic engagement, and building a safe and equitable space. Building an inclusive and diverse space requires an intentional and active effort. Research team members are called on to advance their ongoing training; to move past any preconceived notions of mental health, substance use health, lived/living experience, and of the population of interest; and to do active outreach to maximize inclusivity and diversity in engagement spaces.

## Supplementary Information

Below is the link to the electronic supplementary material.

Supplementary Material 1

Supplementary Material 2

Supplementary Material 3

## Data Availability

The datasets used and/or analyzed during the current study are available from the corresponding author on reasonable request.

## Abbreviation

PLLEX-C: People with lived/living experience and caregivers

## References

[1] Johnston JN, Ridgway L, Cary-Barnard S, Allen J, Sanchez-Lafuente CL, Reive B, et al. Patient oriented research in mental health: matching laboratory to life and beyond in Canada. Res Involv Engagem. 2021;7:1–11. 33902751 10.1186/s40900-021-00266-1 PMC8074277

[2] Towers A-M, Smith N, Allan S, Vadean F, Collins G, Rand S, et al. Care home residents’ quality of life and its association with CQC ratings and workforce issues: the MiCareHQ mixed-methods study. Health Social Care Delivery Res. 2021;9(19):1–188. 34723450

[3] National Institute for Health and Care Research (NIHR). Briefing notes for researchers - public involvement in NHS, health and social care research. Updated 2021 Apr 5 [cited 2025 Jul 25]. Available from: http://nihr.ac.uk/briefing-notes-researchers-public-involvement-nhs-health-and-social-care-research#tab-256876

[4] Sangill C, Buus N, Hybholt L, Berring LL. Service user’s actual involvement in mental health research practices: a scoping review. Int J Ment Health Nurs. 2019;28(4):798–815. 30938019 10.1111/inm.12594

[5] Hawke LD, Sheikhan NY, Rodak T. Lived experience and family engagement in psychiatry research: A scoping review of reviews. Health Expect. 2024;27(3):e14057. 38678591 10.1111/hex.14057 PMC11056206

[6] Carroll P, Dervan A, Maher A, McCarthy C, Woods I, Kavanagh R, et al. Applying patient and public involvement in preclinical research: a co-created scoping review. Health Expect. 2022;25(6):2680–99. 36217557 10.1111/hex.13615 PMC9700145

[7] Sheikhan NY, Kuluski K, McKee S, Hiebert M, Hawke LD. Exploring the impact of engagement in mental health and substance use research: A scoping review and thematic analysis. Health Expect. 2023;26(5):1806–19. 37282732 10.1111/hex.13779 PMC10485342

[8] Hawke LD, Dada-Phillips W, Seiyad H, Orson J, Goldsmith L, Conway S, et al. Best practices guidelines for the engagement of people with lived experience and family members in mental health and substance use health research: A modified Delphi consensus study. Health Expect. 2025;28(1):e70152. 39832210 10.1111/hex.70152 PMC11745228

[9] Lim WM. What is qualitative research? An overview and guidelines. Australasian Mark J. 2025;33(2):199–229.

[10] Greenwood K, Robertson S, Vogel E, Vella C, Ward T, McGourty A, et al. The impact of patient and public involvement in the SlowMo study: reflections on peer innovation. Health Expect. 2022;25(1):191–202. 34585482 10.1111/hex.13362 PMC8849241

[11] Warner G, Baghdasaryan Z, Osman F, Lampa E, Sarkadi A. I felt like a human being’—An exploratory, multi-method study of refugee involvement in the development of mental health intervention research. Health Expect. 2021;24:30–9. 31705620 10.1111/hex.12990 PMC8137488

[12] Sheikhan NY, Hawke LD, Cleverley K, Darnay K, Courey L, Szatmari P, et al. It reshaped how I will do research’: A qualitative exploration of team members’ experiences with youth and family engagement in a randomized controlled trial. Health Expect. 2021;24(2):589–600. 33587827 10.1111/hex.13206 PMC8077141

[13] van Draanen J, Jeyaratnam J, O’Campo P, Hwang S, Harriott D, Koo M, et al. Meaningful inclusion of consumers in research and service delivery. Psychiatr Rehabil J. 2013;36(3):180. 24059630 10.1037/prj0000014

[14] Paul C, Holt J. Involving the public in mental health and learning disability research: can we, should we, do we? J Psychiatr Ment Health Nurs. 2017;24(8):570–9. 28556251 10.1111/jpm.12404

[15] Troya MI, Chew-Graham CA, Babatunde O, Bartlam B, Higginbottom A, Dikomitis L. Patient and public involvement and engagement in a doctoral research project exploring self‐harm in older adults. Health Expect. 2019;22(4):617–31. 31131529 10.1111/hex.12917 PMC6737763

[16] Mawn L, Welsh P, Kirkpatrick L, Webster LA, Stain HJ. Getting it right! Enhancing youth involvement in mental health research. Health Expect. 2016;19(4):908–19. 26202658 10.1111/hex.12386 PMC5152725

[17] Hawke LD, Darnay K, Brown M, Iyer S, Ben-David S, Khaleghi‐Moghaddam M, et al. INNOVATE research: impact of a workshop to develop researcher capacity to engage youth in research. Health Expect. 2020;23(6):1441–9. 32902068 10.1111/hex.13123 PMC7752193

[18] Moltu C, Stefansen J, Svisdahl M, Veseth M. How to enhance the quality of mental health research: service users’ experiences of their potential contributions through collaborative methods. Am J Psychiatric Rehabilitation. 2013;16(1):1–21.

[19] Patterson S, Trite J, Weaver T. Activity and views of service users involved in mental health research: UK survey. Br J Psychiatry. 2014;205(1):68–75. 24723628 10.1192/bjp.bp.113.128637

[20] Goldsmith LP, Morshead R, McWilliam C, Forbes G, Ussher M, Simpson A, et al. Co-producing randomized controlled trials: how do we work together? Front Sociol. 2019;4:21. 33869347 10.3389/fsoc.2019.00021 PMC8022576

[21] Hawke LD, Sheikhan NY, Roberts S, McKee S. Research evidence and implementation gaps in the engagement of people with lived experience in mental health and substance use research: a scoping review. Res Involv Engagem. 2023;9(1):32. 37170357 10.1186/s40900-023-00442-5 PMC10176886

[22] Thomson R, Murtagh M, Khaw FM. Tensions in public health policy: patient engagement, evidence-based public health and health inequalities. BMJ Qual Saf. 2005;14(6):398–400. 10.1136/qshc.2005.014175 PMC1744099 16326781

[23] INVOLVE. Strategies for diversity and inclusion in public involvement: supplement to the briefing notes for researchers. Eastleigh: INVOLVE; 2012.

[24] INVOLVE. Diversity and inclusion: what’s it about and why is it important for public involvement in research? Eastleigh: INVOLVE; 2012.

[25] Tremblay M-C, Bradette-Laplante M, Bérubé D, Brière É, Moisan N, Niquay D, et al. Engaging Indigenous patient partners in patient-oriented research: lessons from a one-year initiative. Res Involv Engagem. 2020;6:1–11. 32760594 10.1186/s40900-020-00216-3 PMC7376932

[26] Lieberson S. Measuring population diversity. Am Sociol Rev. 1969;34(6):850–62.

[27] Madden JM, Foxworth PM, Ross-Degnan D, Allen KG, Busch AB, Callahan MX, et al. Integrating stakeholder engagement with claims-based research on health insurance design and bipolar disorder. Psychiatric Serv. 2021;72(2):186–94. 10.1176/appi.ps.202000177 33167814

[28] Scholz B, Platania-Phung C, Gordon S, Ellis P, Roper C, Bocking J, et al. Very useful, but do carefully: mental health researcher views on Establishing a mental health expert consumer researcher group. J Psychiatr Ment Health Nurs. 2019;26(9–10):358–67. 31343799 10.1111/jpm.12547

[29] Retzer A, Sayers R, Pinfold V, Gibson J, Keeley T, Taylor G, et al. Development of a core outcome set for use in community-based bipolar trials—a qualitative study and modified Delphi. PLoS ONE. 2020;15(10):e0240518. 33112874 10.1371/journal.pone.0240518 PMC7592842

[30] Damon W, Callon C, Wiebe L, Small W, Kerr T, McNeil R. Community-based participatory research in a heavily researched inner city neighbourhood: perspectives of people who use drugs on their experiences as peer researchers. Soc Sci Med. 2017;176:85–92. 28135693 10.1016/j.socscimed.2017.01.027 PMC5438752

[31] Shalowitz MU, Isacco A, Barquin N, Clark-Kauffman E, Delger P, Nelson D, et al. Community-based participatory research: a review of the literature with strategies for community engagement. J Dev Behav Pediatr. 2009;30(4):350–61. 19672162 10.1097/DBP.0b013e3181b0ef14

[32] Jones DE, Lindquist-Grantz R, DeJonckheere M. A review of mixed methods community-based participatory research applications in mental health. J Social Behav Health Sci. 2020;14(1):254–88.

[33] Pandya-Wood R, Barron DS, Elliott J. A framework for public involvement at the design stage of NHS health and social care research: time to develop ethically conscious standards. Res Involv Engagem. 2017;3:1–21. 29062531 10.1186/s40900-017-0058-y PMC5611655

[34] Cohen O. How do we recover? An analysis of psychiatric survivor oral histories. J Humanistic Psychol. 2005;45(3):333–54.

[35] Hamilton CB, Hoens AM, Backman CL, McKinnon AM, McQuitty S, English K, et al. An empirically based conceptual framework for fostering meaningful patient engagement in research. Health Expect. 2018;21(1):396–406. 28984405 10.1111/hex.12635 PMC5750689

[36] O’Brien BC, Harris IB, Beckman TJ, Reed DA, Cook DA. Standards for reporting qualitative research: a synthesis of recommendations. Acad Med. 2014;89(9):1245–51. 24979285 10.1097/ACM.0000000000000388

[37] Staniszewska S, Brett J, Simera I, Seers K, Mockford C, Goodlad S, et al. GRIPP2 reporting checklists: tools to improve reporting of patient and public involvement in research. BMJ. 2017;358. 10.1136/bmj.j3453 PMC5539518 28768629

[38] Campbell S, Greenwood M, Prior S, Shearer T, Walkem K, Young S, et al. Purposive sampling: complex or simple? Research case examples. J Res Nurs. 2020;25(8):652–61. 34394687 10.1177/1744987120927206 PMC7932468

[39] Harris PA, Taylor R, Thielke R, Payne J, Gonzalez N, Conde JG. Research electronic data capture (REDCap)—a metadata-driven methodology and workflow process for providing translational research informatics support. J Biomed Inform. 2009;42(2):377–81. 18929686 10.1016/j.jbi.2008.08.010 PMC2700030

[40] Fereday J, Muir-Cochrane E. Demonstrating rigor using thematic analysis: A hybrid approach of inductive and deductive coding and theme development. Int J Qualitative Methods. 2006;5(1):80–92.

[41] QSR International Pty Ltd. NVivo 12. QSR International; 2020.

[42] Hamilton CB, Hoens AM, McKinnon AM, McQuitty S, English K, Hawke LD, et al. Shortening and validation of the patient engagement in research scale (PEIRS) for measuring meaningful patient and family caregiver engagement. Health Expect. 2021;24(3):863–79. 33729634 10.1111/hex.13227 PMC8235891

[43] Neille J, Penn C. Beyond physical access: a qualitative analysis into the barriers to policy implementation and service provision experienced by persons with disabilities living in a rural context. Rural Remote Health. 2015;15(3):149–62. 26268958

[44] Barendregt C, Van der Poel A, Van de Mheen D. Tracing selection effects in three non-probability samples. Eur Addict Res. 2005;11(3):124–31. 15990429 10.1159/000085547

[45] Woolf SH, Zimmerman E, Haley A, Krist AH. Authentic engagement of patients and communities can transform research, practice, and policy. Health Aff. 2016;35(4):590–4. 10.1377/hlthaff.2015.1512 PMC4868544 27044956

[46] Carter SM, Shih P, Williams J, Degeling C, Mooney-Somers J. Conducting qualitative research online: challenges and solutions. Patient-Patient-Centered Outcomes Res. 2021;14(6):711–8. 10.1007/s40271-021-00528-w PMC8192219 34114170

[47] Bhui K, Bhugra D. Mental illness in black and Asian ethnic minorities: pathways to care and outcomes. Adv Psychiatr Treat. 2002;8(1):26–33.

[48] McIntyre C, Harris MG, Baxter AJ, Leske S, Diminic S, Gone JP, et al. Assessing service use for mental health by Indigenous populations in Australia, Canada, New Zealand and the United States of America: a rapid review of population surveys. Health Res Policy Syst. 2017;15:1–17. 28778208 10.1186/s12961-017-0233-5 PMC5544983

[49] Romanelli M, Hudson KD. Individual and systemic barriers to health care: perspectives of lesbian, gay, bisexual, and transgender adults. Am J Orthopsychiatry. 2017;87(6):714. 29154611 10.1037/ort0000306

[50] Thomas F, Hansford L, Wyatt K, Byng R, Coombes K, Finch J, et al. An engaged approach to exploring issues around poverty and mental health: a reflective evaluation of the research process from researchers and community partners involved in the destress study. Health Expect. 2021;24:113–21. 32449304 10.1111/hex.13065 PMC8137483

[51] Allen D, Cree L, Dawson P, El Naggar S, Gibbons B, Gibson J, et al. Exploring patient and public involvement (PPI) and co-production approaches in mental health research: learning from the PARTNERS2 research programme. Res Involv Engagem. 2020;6(1). 10.1186/s40900-020-00224-3 PMC7507647 32974052

[52] Wadman R, Williams AJ, Brown K, Nielsen E. Supported and valued? A survey of early career researchers’ experiences and perceptions of youth and adult involvement in mental health, self-harm and suicide research. Res Involv Engagem. 2019;5:1–12. 31164992 10.1186/s40900-019-0149-z PMC6489170

[53] Burnette CE, Sanders S. Trust development in research with Indigenous communities in the United States. Qualitative Rep. 2014;19(22).

[54] Bowen FR, Epps F, Lowe J, Guilamo-Ramos V. Strategies to restore trust in research among historically marginalized communities. Available at SSRN 4065750. 2022. 10.1016/j.outlook.2022.06.006 36229259

[55] Woodall A, Morgan C, Sloan C, Howard L. Barriers to participation in mental health research: are there specific gender, ethnicity and age related barriers? BMC Psychiatry. 2010;10:1–10. 21126334 10.1186/1471-244X-10-103 PMC3016310

[56] University of West England. Public involvement in research: guidelines for good practice. 2011 [cited 2025 July 3]. Available from: http://hls.uwe.ac.uk/suci/Data/Sites/1/heifposter.pdf

[57] Witteman HO, Chipenda Dansokho S, Colquhoun H, Fagerlin A, Giguere AM, Glouberman S, et al. Twelve lessons learned for effective research partnerships between patients, caregivers, clinicians, academic researchers, and other stakeholders. J Gen Intern Med. 2018;33:558–62. 29327211 10.1007/s11606-017-4269-6 PMC5880766

[58] Black A, Strain K, Wallsworth C, Charlton S-G, Chang W, McNamee K, et al. What constitutes meaningful engagement for patients and families as partners on research teams? J Health Serv Res Policy. 2018;23(3):158–67. 29504424 10.1177/1355819618762960 PMC6041763

[59] AbSPORU Patient Engagement Platform. Patient engagement in health research: a How-to guide for patients. Alberta SPOR SUPPORT Unit; 2018.

[60] Canadian institutes of health research. Strategy for patient-oriented research - patient engagement framework. 2014 [updated May 27, 2019; cited 2025 June 26]. Available from: https://www.cihr-irsc.gc.ca/e/48413.html#a7

[61] Frisch N, Atherton P, Doyle-Waters MM, MacLeod ML, Mallidou A, Sheane V, et al. Patient-oriented research competencies in health (PORCH) for researchers, patients, healthcare providers, and decision-makers: results of a scoping review. Res Involv Engagem. 2020;6:1–14. 32055415 10.1186/s40900-020-0180-0 PMC7011284

[62] Ross LE, Pilling M, Voronka J, Pitt K-A, McLean E, King C, et al. I will play this tokenistic game, I just want something useful for my community’: experiences of and resistance to harms of peer research. Crit Public Health. 2023;33(5):735–46.

[63] Richards DP, Bowden J, Gee P, Haagaard A, Kothari A, McKinnon A, et al. The ultimate power play in research-partnering with patients, partnering with power. Res Involv Engagem. 2025;11:65. 40528262 10.1186/s40900-025-00745-9 PMC12172280

[64] Kamberelis G, Dimitriadis G. Focus groups: strategic articulations of pedagogy, politics, and inquiry. 2005.

[65] Fern EF. The use of focus groups for Idea generation: the effects of group size, acquaintanceship, and moderator on response quantity and quality. J Mark Res. 1982;19(1):1–13.

[66] Carey A. The group effect in focus groups: Planning, implementing and interpreting focus group research. Critical issues in qualitative research methods. Thousand Oaks: Sage; 1994. pp. 225–41.

[67] Morgan DL. Focus groups. Ann Rev Sociol. 1996;22(1):129–52.

