# Supplementary material for: “Inclusivity requires an active effort”: building an inclusive and diverse space when engaging people with lived and living experience and caregivers in mental health and substance use health research
Source: Res Involv Engagem. 2025 Oct 29;11:129. doi: 10.1186/s40900-025-00798-w (PMC12574087; doi:10.1186/s40900-025-00798-w)
Supplement: Supplementary file 1 — Supplementary Material 1 [file 40900_2025_798_MOESM1_ESM.docx]

**Inclusivity and accessibility**

**Semi-structured interview guide**

# **Introduction& Reminder**

[Icebreaker: Share your alias name. What would you pick if you could choose your own superpower? OR If you could be an animal for a day, which would you choose?]

**Reminder:** Voluntary participation**,** Confidentiality & privacy, Audio recording & Consent, WebEx Etiquette

Before we begin, I would like to repeat some reminders regarding your participation in this study.

1. I would like to remind you that participating in the study is voluntary. It is your choice whether you decide to take part in this study or not. If you do decide to participate, you can change your mind and withdraw at any time. No matter what you decide, it will not affect the care or any other services that you receive at CAMH. If you want to leave today’s meeting early, please message me or our back-up facilitator.
2. The information you provide during this meeting will be kept confidential according to CAMH policies and the extent permitted by law. There are some special circumstances in which the researchers may need to share information about you. For example, they may need to disclose information about you if they are worried about your safety or the safety of someone else, if our files are subpoenaed by a court of law, or if public health laws require that health professionals report a communicable disease. In the unlikely case that this happens, your information may be shared with the police, healthcare, protective services or mandated government departments and agencies (such as local public health authorities).
3. We also remind you that our discussion today will be recorded and the recording will be transcribed. I would like to make sure that we have your consent to record this section. *(Pause to see if anyone objects or have questions)*
4. Last but not least, some reminders about group discussion etiquette:
   1. Please remember that you are not allowed to have anyone else join the call.
   2. We would prefer it if you kept your camera on during the session if possible, but this is not required.
   3. To ensure your confidentiality, please don’t display your real name on WebEx. Please display the alias or pseudonym you have chosen in the previous consent meeting.
   4. You must not record the session or take photos/screenshot.
   5. Please be respectful of other participants throughout the session.
   6. Feel free to message the host if you have questions, concerns, or have to leave the meeting early. We may try to contact you if you happen to leave the group without any message.

# **Preamble**

Today, we’re going to talk about your experience being part of a research team as an advisor, collaborator, co-researcher, or in another role. We’re not talking about being involved as a research participant, but rather being part of the research team. We want to know about inclusivity and accessibility. Inclusivity means making sure our research team activities include a lot of different people who can each contribute in a way that works for them. We’re going to talk about improving inclusivity and accessibility.

**[Start recording]**

1. Tell us about when you were a member of a research team. How did it go for you? [*introduction*] – 5 minutes
2. When you were a member of a research team, what other kinds of people were there with you? [*introduction*] – 10 minutes **(If they ask to expand…add examples, background characteristics (LE, family) or levels of engagement experience).**
   1. How inclusive did the space feel?
   2. Were there members with different perspectives? What was that like?
   3. What kinds of people were missing in the room?
3. People with lived experience and family members tell us that it’s important for research teams to be inclusive. Based on your lived experience, what does inclusivity mean to you? 10 minutes
   1. How has inclusivity affected the way you have been able to be involved in research?
   2. How has the lack of inclusivity affected the way you have been involved in research?
   3. How does inclusivity affect the research?
   4. How does the lack of inclusivity affect the research?
4. Now, remember or imagine a time when you were a member of a research team and it felt like a diverse and inclusive space. What did that look like? 10 minutes
   1. How did you know it was diverse and inclusive?
   2. How did diversity and inclusivity influence the research?
   3. What could have made it more diverse and inclusive?
   4. When should teams be talking about diversity and inclusivity?
5. Now, remember or imagine a time when you were a member of a research team and it felt like it was not a diverse and inclusive space. What did that look like? 10 minutes
   1. What kinds of perspectives were missing?
   2. How did the lack of diversity and inclusivity influence the research?
   3. What could have made it more diverse and inclusive?

[5-minute break]

Accessibility can include things like helping people physically access a space and helping to make sure they can understand the material. Accessibility is about removing barriers and providing people what they need to contribute fully to the project, like providing childcare or respite, addressing transportation barriers and providing communication support.

1. Now, let’s think about accessibility. Based on your lived experience, what makes it difficult to work as a member of a research team?
   1. What makes a research meeting more accessible?
   2. What barriers have you experienced? How could they be addressed?
2. Think back to a time when you were a member of a research team and it was very accessible to you and other people. What did that look like? 10 minutes
   1. Was it online or in person?
   2. How was it accessible to you and others?
   3. What could have made it more accessible?
   4. How did accessibility affect the research?
3. Think back to a time when you were a member of a research team and it was NOT very accessible to you or other people. What did that look like? 10 minutes
   1. Was it online or in person?
   2. How was it not accessible to you and others?
   3. What could have made it more accessible?
   4. How did inaccessibility affect the research?
4. How do you think technology can be used to make research engagement more accessible? 10 minutes
   1. What kinds of technology tools or platforms should we use?
   2. What can get in the way of using technology?
   3. How can we best support people when using new technology?
5. How are in-person and virtual meetings different in terms of accessibility?
   1. Who finds in-person meetings more accessible? Why?
   2. Who finds virtual meetings more accessible? Why?
   3. What about hybrid meetings?
6. Can you imagine a case when someone’s access needs conflict with someone else’s? For example, one person may need to be off camera, but another may require lip reading.
   1. Has anyone experienced this? What were the conflicting needs?
   2. How should we manage conflicting needs?
7. What is your experience with creating terms of reference documents to guide research teams?

**Definition:** (scope, objectives, deliverables, roles, responsibilities, timeline, and other key details of a project or task)

- 1. How have these affected the accessibility of a group?
  2. How have these helped manage conflicting needs?

1. Is there anything else you want to tell us about being a member of a research team? [*conclusion*] 5 minutes

[Closure, thank participants, inform them of next steps for the honoraria.]
