# Supplementary material for: “Inclusivity requires an active effort”: building an inclusive and diverse space when engaging people with lived and living experience and caregivers in mental health and substance use health research
Source: Res Involv Engagem. 2025 Oct 29;11:129. doi: 10.1186/s40900-025-00798-w (PMC12574087; doi:10.1186/s40900-025-00798-w)
Supplement: Supplementary file 3 — Supplementary Material 3 [file 40900_2025_798_MOESM3_ESM.docx]

GRIPP-2 short form checklist

| Section & topic | Item | Reported on page No |
| --- | --- | --- |
| 1: Aim | To draw on the lived/living expertise, learned expertise, and knowledge base of PLLEX-C team members. Their perspectives helped to close the gap between the research and their experiences of mental health and substance use health, which helped inform our study on how to make research engagement spaces more inclusive and diverse. | 10 |
| 2: Methods | A PLLEX-C Working Group was created consisting of three to six members, depending on the project stage. At study initiation, the research team developed a terms of reference agreement, and a consensus meeting time was decided by poll. We launched the virtual Working Group meetings in April 2024 and concluded them in July 2025. There were 20 meetings held from the design of the overarching study to the finalization of the current manuscript. Meetings were initially 1 hour in duration; however, they were extended to 2 hours based on need. They were held either monthly or semi-monthly, with the team working on this project and other related sub-projects. Compensation was provided at an hourly rate for meeting attendance and study-related tasks completed outside of meeting times based on a flexible pre-specified allotment of time for activities. PLLEX-C also indicated their method to receive honoraria. Several accessibility supports were provided including alternative software options, basic training of the WebEx system, troubleshooting technical difficulties, allowing multiple ways to contribute (e.g., chat, email, or verbal communication/feedback), and allowing Working Group members to participate with their camera off during team meetings. | 10 |
| 3: Study results | PLLEX-C were engaged at all levels of the research process from design to knowledge translation. PLLEX-C reviewed research ethics materials (e.g., the interview guide and study flyer). One member helped develop the codebook. All members analyzed initial codes and themes, chose quotes for inclusion, co-authored the manuscript, helped with grant and conference applications/ submissions, and suggested relevant research questions for future exploration. | 10-11 |
| 4: Discussion and conclusions | The PLLEX-C engagement approach promoted trust and collaboration. The research was rooted in real-world experience, equity, diversity, and inclusivity. Strategies for recruitment and study tools had greater relevance to the participants. Meetings were initially held on the WebEx system, but were moved to Zoom to accommodate Working Group familiarity and preference. We also held ongoing informal conversations about how engagement was going, which supported iterative improvements to our engagement approach. Engagement was formally evaluated using the Patient Engagement in Research Scale near the end of the project period (38). | 11 |
| 5: Reflections and critical perspective | PLLEX-C were able to build personal and professional skills through their involvement in the Working Group. This included a better understanding of qualitative research methods, as well as learning how to address the needs of the project and interpersonal interactions in a group environment. Their lived and living experiences were respected and valued and their experiences and perspectives were affirmed. Scientists and research staff on the team grew their understanding of the structural components of the work, which facilitated reflection and co-learning. | 11 |
